# Eccentric Isokinetic Rehabilitation for Chronic Lateral Epicondylitis in Female Swimmers: A Randomized Controlled Trial of Bilateral Neuromuscular Adaptations and Functional Performance

**DOI:** 10.3390/medicina62030494

**Published:** 2026-03-05

**Authors:** Wissem Dhahbi, Hatem Ghouili, Halil İbrahim Ceylan, Nessrine Adhadhi, Souhail Bchini, Manel Bessifi, Nagihan Burçak Ceylan, Valentina Stefanica, Nejmeddine Ouerghi, Nadhir Hammami

**Affiliations:** 1 Research Unit “Sport Sciences, Health and Movement” (UR22JS01), High Institute of Sport and Physical Education of Kef, University of Jendouba, Kef 7100, Tunisia; wissem.dhahbi@gmail.com (W.D.); hatemghouili@gmail.com (H.G.); adhadhi.nesrine2017@gmail.com (N.A.); souhail.bchini@gmail.com (S.B.); manelbessifii@gmail.com (M.B.); najm_ouerghi@hotmail.com (N.O.); nadhir.hammami@issepkef.u-jendouba.tn (N.H.); 2 Training Department, Police College, Qatar Police Academy, Doha 12903, Qatar; 3 Physical Education of Sports Teaching Department, Faculty of Sports Sciences, Atatürk University, 25240 Erzurum, Türkiye; 4 Graduate Education Institute, Bayburt University, 69000 Bayburt, Türkiye; burcaksehitoglu@gmail.com; 5 Department of Physical Education and Sport, Faculty of Sciences, Physical Education and Informatics, National University of Science and Technology Politehnica Bucharest, Pitești University Center, 060042 Pitești, Romania; 6 Faculty of Medicine of Tunis, University of Tunis El Manar, Rabta Hospital, LR99ES11, Tunis 1007, Tunisia

**Keywords:** athletic injuries, biomechanical phenomena, muscle contraction, physical therapy modalities, rehabilitation, resistance training, tennis elbow, upper extremity

## Abstract

*Background and Objectives*: This study investigated the efficacy of eccentric isokinetic muscle strengthening versus passive motion protocols on neuromuscular function and performance capacity in female swimmers with chronic lateral epicondylitis. *Materials and Methods*: Twenty-five swimmers (age 46.1 ± 3.1 years) with lateral epicondylitis exceeding three months’ duration completed a randomized controlled trial comparing eccentric training in Controlled Active Motion mode (experimental group (EG), *n* = 13) against passive motion in Continuous Passive Motion mode (control group (CG), *n* = 12). Both groups performed 18 supervised sessions over six weeks (60°/s angular velocity, progressive loading 1–12 sets × 5 repetitions). Bilateral concentric peak torque of elbow extensors and flexors constituted the primary outcomes. Secondary measures included push-up performance, explosive power assessed by the Seated Medicine Ball Chest Push Test, and goniometric range of motion. Linear mixed-effects models and analysis of covariance with baseline adjustment were employed. *Results*: Eccentric training produced side-specific strength adaptations in elbow flexors (confirmed interaction: F_1,23_ = 8.56, *p* = 0.008, *η*_p_^2^ = 0.271), with the experimental group demonstrating balanced bilateral gains, whereas the control group exhibited asymmetric responses favoring the non-dominant limb. EG demonstrated superior functional gains: push-up repetitions increased 4.15 ± 1.77 versus 2.17 ± 1.27 in CG (adjusted difference = 3.21 repetitions, 95% CI [1.52, 4.90], *p* = 0.001, *d* = 1.31), while explosive power improved 0.32 ± 0.09 m versus 0.10 ± 0.06 m (adjusted difference = 0.35 m, 95% CI [0.25, 0.45], *p* < 0.001, *d* = 1.20). Range of motion remained unchanged across groups (all *p* > 0.65). *Conclusions*: Eccentric isokinetic strengthening confers substantial advantages over passive motion protocols for restoring upper-body muscular endurance and ballistic force production in swimmers with lateral epicondylitis, supporting its integration into rehabilitation frameworks for the management of tendinopathy.

## 1. Introduction

The human elbow constitutes a biomechanical link between the brachium and the forearm, controlling upper extremity reach length and hand orientation through the coordinated function of four articulating joints. This anatomical complexity enables precise motor control but simultaneously creates vulnerability to overuse pathology when subjected to repetitive loading patterns characteristic of athletic training [1], particularly when biomechanical factors such as altered muscle activation patterns or joint kinematics are present [2,3]. Lateral epicondylitis is a prevalent musculoskeletal disorder affecting approximately 1–3% of the general population annually, with incidence rates as high as 15% among occupational groups performing repetitive upper extremity tasks [4]. The condition is the predominant cause of elbow pain and is associated with significant functional limitation in activities requiring repetitive pronosupination of the forearm and elbow extension. Despite swimming’s status as the second-largest participation sport at the Olympic Games and its global popularity across the lifespan [1], elbow pain among aquatic athletes has received insufficient research attention. Popular perception focuses on the risk of swimming-related injury to the shoulder, lower back, knee, and ankle, obscuring recognition of the prevalence of elbow pathology. Regional research disparities compound this neglect, with systematic investigation of swimmer’s elbow pain initiated only within the past decade, predominantly in North American institutions [5].

Contemporary understanding of the pathophysiology of lateral epicondylitis has evolved substantially from historical inflammatory models toward recognition of degenerative tendinosis characterized by failed healing responses [6]. Histopathological investigations consistently demonstrate disordered collagen architecture, increased type III collagen deposition, neovascularization with accompanying nociceptive nerve ingrowth, and accumulation of glycosaminoglycans within affected extensor carpi radialis brevis tendons [7]. These structural alterations compromise tensile strength and load-bearing capacity, perpetuating a cycle of microtrauma accumulation during repetitive activities. The absence of inflammatory cell infiltrates in chronic presentations has prompted a shift in therapeutic paradigms from anti-inflammatory interventions toward mechanotherapy approaches that stimulate adaptive tendon remodeling through controlled mechanical loading [8]. This mechanobiological framework posits that appropriately dosed tensile stimuli activate mechanotransduction pathways regulating tenocyte metabolism, collagen synthesis, and extracellular matrix organization.

Conservative management modalities for lateral epicondylitis encompass diverse interventions, with Chard and Hazleman [9] documenting more than 40 distinct treatment approaches. Despite this therapeutic abundance, comparative effectiveness data remain limited and heterogeneous, with evidence quality frequently compromised by methodological limitations including inadequate sample sizes, brief follow-up periods, and failure to stratify participants by symptom chronicity or severity [10]. Corticosteroid injections provide short-term symptom relief but demonstrate inferior long-term outcomes compared to expectant management, with elevated recurrence rates and potential for tendon structural deterioration. The theoretical effectiveness of anti-inflammatory treatments appears doubtful, given current pathophysiological understanding emphasizing degenerative rather than inflammatory processes. Conversely, substantial empirical evidence supports eccentric exercise protocols across multiple tendinopathy sites.

Eccentric exercise protocols have robust evidence supporting their use in the management of chronic tendinopathy, particularly affecting the Achilles and patellar tendons. Eccentric strengthening loads the musculotendinous unit to induce hypertrophy and increased tensile strength, reducing strain on the tendon during movement [11,12]. The training paradigm conditions tissue to withstand forces greater than those encountered during inciting activities [13]. Contemporary mechanistic theories propose that eccentric strengthening reduces pathological neovascularization, which is believed to be causative in painful tendinopathies, and investigations have documented significant normalization of tendon size following 12-week eccentric protocols [14,15]. Meta-analytic evidence demonstrates that eccentric training combined with adjuvant therapies significantly improves pain intensity (standardized mean difference −0.63, 95% confidence interval [−0.90, −0.36]) and muscle strength (standardized mean difference 1.05, 95% confidence interval [0.78, 1.33]) compared to adjuvant therapies alone in lateral epicondylitis populations [16]. However, existing investigations exhibit substantial methodological heterogeneity in exercise parameters, including contraction intensity, angular velocity, volume prescription, and progression strategies, with most studies employing simple resistance equipment, which precludes precise quantification of eccentric loading characteristics.

Isokinetic dynamometry provides advantages for tendinopathy rehabilitation through velocity control, force quantification, elimination of gravitational compensation, and progressive loading. This widely used method for assessing limb strength [1] assesses force production while modulating neural control and enhancing joint stability and coordination [17,18], with established relationships to athletic performance [19,20]. Croisier et al. [21] demonstrated that isokinetic eccentric training for lateral epicondylitis (30% intensity at 30°/s progressing to 80% at 90°/s over nine weeks) produced superior outcomes compared to non-strengthening modalities. Despite such findings, isokinetic protocols remain underutilized in the tendinopathy rehabilitation literature.

Swimming populations constitute a salient yet neglected demographic in research on lateral epicondylitis. Freestyle stroke biomechanics impose asymmetric loading through unilateral breathing patterns: rotation toward the preferred breathing side generates differential glenohumeral and elbow joint forces between limbs [2,3,22], with the breathing-side arm experiencing prolonged abduction and altered scapulohumeral rhythm during inhalation phases. Repetitive asymmetric loading may predispose one limb to extensor carpi radialis brevis tendon microtrauma while inducing compensatory neuromuscular adaptations in the contralateral limb. Swimmers demonstrate sport-specific adaptations, including enhanced muscle endurance, bilateral coordination demands that exceed those of unilateral racquet sports, and distinct motor control strategies that may influence rehabilitation responses. Breathing-side preference may thus determine laterality-dependent strength profiles and treatment adaptations. Although alternative loading strategies, such as slow and heavy resistance training, have demonstrated effectiveness in lower-limb tendinopathies, eccentric exercise remains one of the most studied and clinically implemented approaches for the rehabilitation of upper-limb tendinopathies [23,24]. No prior investigation has employed isokinetic muscle-strengthening programs as therapeutic interventions for swimmers with lateral epicondylitis. Although eccentric exercise represents an established tendinopathy intervention [11,12], swimming populations remain underrepresented in this literature, and bilateral strength adaptations reflecting sport-specific laterality patterns have received limited systematic investigation.

This investigation addresses these substantive gaps by evaluating eccentric isokinetic muscle strengthening in Controlled Active Motion mode versus passive motion protocols in Continuous Passive Motion mode among female swimmers with chronic lateral epicondylitis. The study employs a comprehensive outcome assessment that includes bilateral concentric peak torque measurements, functional performance tests that reflect sport-specific demands, and a passive range-of-motion evaluation. We hypothesized that eccentric training would produce greater improvements in bilateral strength profiles, functional capacity, and limb symmetry than passive motion interventions. At the same time, both protocols would demonstrate equivalent effects on passive joint mobility.

## 2. Materials and Methods

### 2.1. Participants

Thirty female master swimmers (age 40–50 years) with chronic lateral epicondylitis were recruited from Kuwait’s National Swimming Academy. An age restriction targeted the peak incidence range of lateral epicondylitis [4], while maintaining competitive training volume characteristic of Master’s athletes, thereby minimizing age-related neuromuscular confounding. Eligibility criteria comprised: (1) elbow pain during swimming for ≥30 days pre-enrollment; (2) lateral epicondylitis diagnosis exceeding three months’ duration per standardized criteria [10]; (3) minimum three years’ competitive swimming experience; (4) active training status. Exclusion criteria encompassed recent musculoskeletal injuries within the preceding three months (fractures, impingements, surgical interventions), concomitant neurological conditions (supinator syndrome, compartment syndrome of musculus anconeus, cervical radiculopathy), inflammatory joint diseases, fibromyalgia, and current engagement in physical therapy for upper extremity conditions. Participants discontinued involvement if they missed more than two weekly sessions, sustained acute injuries during the intervention period, or experienced pain exacerbation requiring medical withdrawal.

Randomization to experimental (EG, *n* = 15) or control (CG, *n* = 15) groups was performed using a computer-generated sequence with allocation concealment via sequentially numbered, opaque, sealed envelopes. The study employed a single-blind design in which outcome assessors remained unaware of group allocation. In contrast, participants and intervention administrators could not be blinded because of the nature of the isokinetic training modalities. Before participant recruitment, an a priori sample size calculation was conducted using G*Power software (version 3.1.9.4; University of Kiel, Kiel, Germany) [25] for a repeated-measures ANOVA with a within-between interaction. The calculation was based on a statistical power of 0.95, an alpha level of 0.05, and a large effect size (f = 0.55), as reported in previous studies [26,27]. The analysis indicated that a minimum sample size of 14 participants per group was required.

During the six-week intervention period, five participants discontinued the protocol: two from EG (one lost to follow-up; one protocol non-compliance, defined as exceeding two consecutive absences) and three from CG (two lost to follow-up; one pain exacerbation requiring medical withdrawal). Final per-protocol analysis included 13 participants in the EG and 12 in the CG (total *n* = 25, retention rate 83.3%). Baseline characteristics of completers versus non-completers were compared using independent-samples *t*-tests to assess potential attrition bias.

The Institutional Review Board of the Higher Institute of Sport and Physical Education of Kef (ISSEP-KEF), University of Jendouba, Jendouba, Tunisia, granted ethical approval prior to participant enrollment (ISSEPK-117/2024; 11 December 2024). The trial was subsequently registered in the Pan African Clinical Trial Registry (PACTR202602776509124) in accordance with the Declaration of Helsinki principles [28]. Registration occurred after study commencement because the PACTR requirement was identified following initiation of enrollment; all pre-specified primary and secondary outcomes were registered without modification. Following a comprehensive briefing on objectives, procedures, risks, and confidentiality protocols, participants and legal guardians provided written informed consent. Voluntary participation rights, including the right to penalty-free withdrawal, were explicitly communicated. Data security was maintained through anonymization and encrypted digital storage [29].

### 2.2. Study Design and Interventions

**Figure 1:**
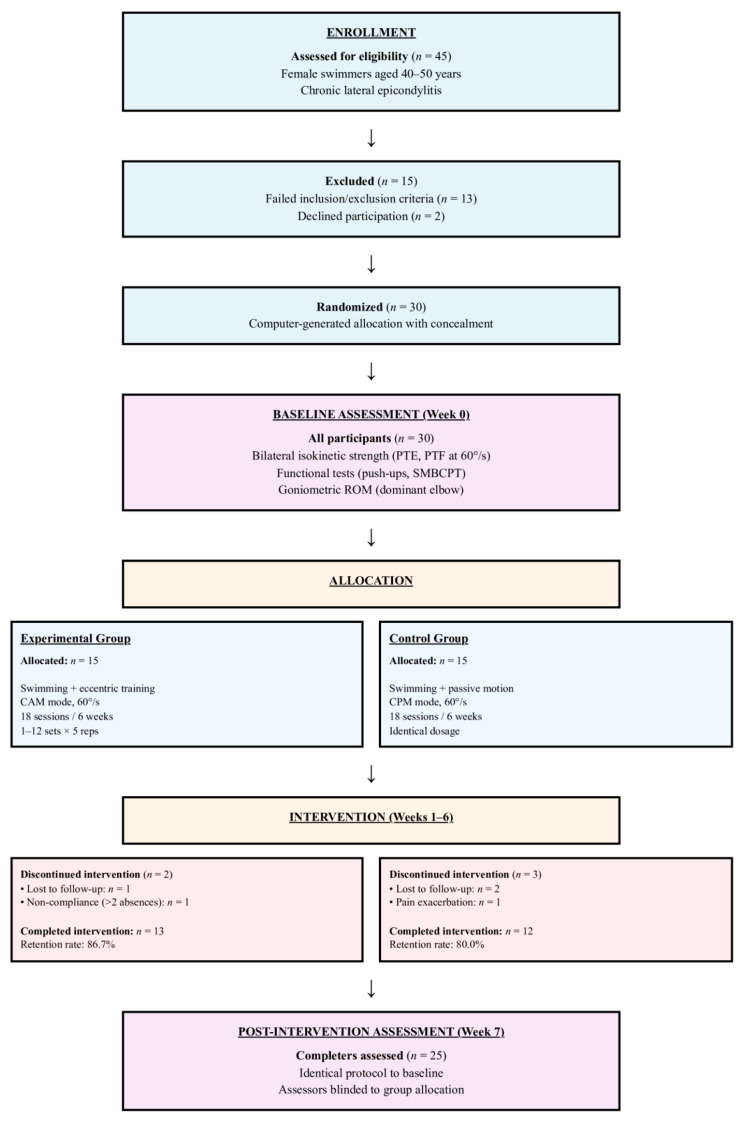
CONSORT flow diagram: Participant progression through randomized controlled trial of isokinetic eccentric strengthening in swimmers with lateral epicondylitis. CAM = Controlled Active Motion; CPM = Continuous Passive Motion.

This randomized controlled trial employed a parallel-group design with pre-intervention and post-intervention assessments separated by a six-week training period. Both groups maintained their regular swimming training schedules. They completed 18 supervised isokinetic strengthening sessions (three sessions per week, with a minimum 48 h inter-session recovery period), using an Isoforce dynamometer (TUR GmbH, Berlin, Germany) (Figure 1). EG performed eccentric isokinetic muscle strengthening in Controlled Active Motion (CAM) mode, characterized by maximal voluntary eccentric contractions of elbow extensors and flexors at constant 60°/s angular velocity across a 10–125° range of motion. CG performed identical exercises in Continuous Passive Motion (CPM) mode, in which the dynamometer-controlled limb was displaced at 60°/s with submaximal participant effort. Progression was volume-based: session 1 comprised 1 set of 5 repetitions, increasing by 1 set per session until 12 sets of 5 repetitions (session 12), maintained through session 18. Contraction intensity remained maximal (EG) or submaximal (CG) throughout; angular velocity was fixed at 60°/s for both groups. Each session began with a 10 min warm-up on an elliptical trainer at 60–70% of predicted maximum heart rate, followed by dynamic stretching of the biceps brachii, brachioradialis, and wrist flexor–extensor groups [30].

### 2.3. Outcome Measures

#### 2.3.1. Isokinetic Muscle Strength Assessment

Bilateral concentric peak torque of elbow extensors (PTE) and flexors (PTF) constituted the primary outcomes, assessed at 60°/s using the Isoforce dynamometer, which demonstrates high test–retest reliability (ICC = 0.80–0.90) for eccentric elbow strength evaluations [31]. Participants assumed a supine position with glenohumeral abduction at 45°, with the upper arm supported on a stabilization pad and the thoracic and pelvic regions secured with nonelastic straps. The dynamometer’s rotational axis was aligned with the lateral epicondyle of the humerus, and participants grasped the input adapter handle with the forearm in neutral rotation. In accordance with the manufacturer’s specifications, gravity correction was not applied. The testing order for the right and left extremities was randomized to minimize learning effects. Participants completed three submaximal warm-up repetitions at 50% perceived effort, followed by five maximal concentric contractions for each limb. Peak torque values (Newton-meters, Nm) were extracted from the repetition demonstrating the highest force production, with bilateral measurements recorded to account for potential asymmetries related to stroke biomechanics and dominant-side breathing patterns in freestyle swimming [22].

#### 2.3.2. Functional Performance Tests

Upper extremity functional capacity was quantified using two standardized field tests. The push-up test assessed muscular endurance by recording the maximum number of continuous repetitions performed with proper form (elbows flexing to 90°, trunk maintained in neutral alignment) until volitional fatigue, technical failure, or pain-induced termination [32]. The Seated Medicine Ball Chest Push Test (SMBCPT) quantified explosive upper-body power [33]. Participants assumed a seated position (back against the wall, legs extended, feet hip-width apart) while holding a 2 kg medicine ball at chest level. Following warm-up throws (50% and 100% perceived effort), three maximal-effort trials were executed with instructions to project the ball anteriorly while maintaining spinal-wall contact and elbow adduction. Throw distance was measured from the wall to the ball’s initial ground contact point, with the greatest distance across three trials recorded.

#### 2.3.3. Clinical Examination

Elbow joint range of motion was assessed for the dominant upper extremity (defined as the breathing-side arm in freestyle stroke) using a standard universal goniometer. With participants’ supine and upper extremity relaxed, the goniometer’s fulcrum was centered over the lateral epicondyle, the stationary arm aligned with the longitudinal axis of the humerus, and the mobile arm aligned with the radial shaft. Passive flexion and extension amplitudes were measured in degrees, with the examiner moving the joint through its available range while monitoring for compensatory movements or pain responses [34]. All assessments were performed by a single trained examiner who was blinded to group allocation, and measurement reliability was established during a pilot phase (ICC > 0.90 for all goniometric measurements).

### 2.4. Procedures

One week before baseline assessments, participants attended a familiarization session to minimize learning effects and establish measurement reliability. Anthropometric data, including height (measured via stadiometer to the nearest 0.1 cm), body mass (assessed using an OMRON BF-212 bioelectrical impedance analyzer, Kyoto, Japan), body mass index (calculated as mass/height^2^, kg/m^2^), and body fat percentage, were recorded. Forearm length was measured bilaterally from the lateral epicondyle to the ulnar styloid process using a non-deformable anthropometric tape. Limb dominance was determined by self-reported hand preference for writing and throwing. Breathing-side preference was assessed by observing three freestyle stroke cycles during warm-up, recording the side toward which head rotation occurred during inhalation.

Pre-intervention and post-intervention testing sessions followed an identical protocol. Following a standardized 10 min warm-up on an elliptical trainer and dynamic stretching routine, participants completed assessments in the following sequence: (1) bilateral isokinetic strength testing (randomized limb order), (2) push-up test, (3) SMBCPT, and (4) goniometric range of motion measurements. Inter-test rest periods of five minutes were implemented to minimize fatigue effects. All testing occurred at consistent times of day (±2 h) to control for circadian influences on neuromuscular performance [35]. Throughout the six-week intervention, session attendance, pain responses (assessed using a numerical rating scale), and adverse events were documented (Table 1).

### 2.5. Statistical Analysis

Statistical analyses were conducted using SPSS version 28.0 (IBM Corporation, Armonk, NY, USA) and R version 4.3.0 [36] with the lme4, emmeans, and effectsize packages. Descriptive data are presented as mean ± standard deviation. Distributional assumptions were evaluated using Shapiro–Wilk tests and Q-Q plots. Homogeneity of variance was assessed via Levene’s test. All outcomes satisfied parametric assumptions (Shapiro–Wilk *p* > 0.05; Levene’s *p* > 0.05; detailed results in Table 2). Baseline anthropometric characteristics were compared between groups using independent-samples *t*-tests.

For bilateral isokinetic strength outcomes (PTE and PTF), linear mixed-effects models were specified to account for repeated-measures (pre–post) and within-subject clustering (right–left limbs). The model included fixed effects for Group (EG vs. CG), Time (pre vs. post), Side (right vs. left), and all two-way and three-way interactions, with random intercepts for participants. Restricted maximum likelihood estimation was employed, and covariance structures were compared using the Akaike Information Criterion. The primary hypothesis tested the Group × Time interaction, representing differential pre-to-post changes between groups. Degrees of freedom were approximated using Satterthwaite’s method.

For unilateral functional outcomes (push-ups, SMBCPT, joint range of motion), analysis of covariance (ANCOVA) models examined post-intervention scores with Group as the fixed factor and baseline scores as covariates, controlling for regression to the mean [36]. Model assumptions were verified through residual diagnostics. Influential observations were identified using Cook’s distance (threshold > 4/25 = 0.16) and standardized difference in beta estimates (DFBETAS; threshold > 2/√25 = 0.40), with sensitivity analyses performed excluding outliers.

Effect sizes were calculated as partial eta-squared (*η*_p_^2^) for ANOVA effects and Cohen’s d for pairwise contrasts, using pooled baseline standard deviations. Magnitude thresholds for standardized differences followed established conventions: small (0.2), moderate (0.6), large (1.2), very large (2.0), and extremely large (4.0) [37]. Intraclass correlation coefficients (ICC [2,1]) with 95% confidence intervals were computed for the random-effects model to quantify between-subject variability. The co-primary outcomes were bilateral PTE and PTF, assessed via Group × Time × Side interactions in mixed-effects models. Bonferroni correction for two co-primary endpoints established α = 0.025 per test (family-wise error rate α = 0.05). Secondary outcomes (push-ups, SMBCPT, ROM) underwent Holm–Bonferroni sequential correction within their outcome family.

Multiple comparison adjustments employed the Holm–Bonferroni sequential procedure for secondary outcomes, maintaining the family-wise error rate of α = 0.05. Primary outcomes (PTE and PTF) were tested at Bonferroni-corrected α = 0.025 each. All tests were two-sided. Exact *p*-values are reported to three decimal places for *p* ≥ 0.001 and in scientific notation otherwise, avoiding inequality symbols that complicate meta-analytic integration [37]. Statistical significance was determined at *p* < 0.05 for secondary outcomes and *p* < 0.025 for co-primary endpoints.

## 3. Results

### 3.1. Participant Characteristics and Baseline Comparisons

Twenty-five female swimmers with chronic lateral epicondylitis completed the six-week intervention (EG *n* = 13, CG *n* = 12). Independent-samples *t*-tests confirmed baseline equivalence for anthropometric variables (Table 3) and primary outcomes (Table 4). Between-group comparisons confirmed baseline equivalence for bilateral extensor peak torque (right: t_23_ = 1.48, *p* = 0.151; left: t_23_ = 0.92, *p* = 0.367), right flexor peak torque (t_23_ = 1.67, *p* = 0.108), push-up capacity (t_23_ = 1.58, *p* = 0.127), SMBCPT distance (t_23_ = 1.47, *p* = 0.154), and ROM (flexion: t_23_ = 0.26, *p* = 0.798; extension: t_23_ = 0.03, *p* = 0.976), confirming successful randomization for these variables. A statistically significant pre-intervention difference was detected for left flexor peak torque (t_23_ = 3.21, *p* = 0.004), with EG demonstrating higher baseline values (0.45 ± 0.14 Nm) than CG (0.30 ± 0.08 Nm); this variable was therefore retained as a covariate in all mixed-effects models to prevent confounding of treatment effects by pre-existing bilateral asymmetry.

Limb dominance and breathing-side preference distributions were similar between groups. EG comprised 12 right-hand dominant (92.3%) and one left-hand dominant participant (7.7%); CG comprised 11 right-hand dominant (91.7%) and one left-hand dominant (8.3%). Breathing-side preference (assessed during freestyle stroke) revealed 11 right-side breathers (84.6%) in EG and 10 right-side breathers (83.3%) in CG. Chi-square tests confirmed no between-group differences in dominance (χ^2^_1_ = 0.00, *p* = 0.95) or breathing preference (χ^2^_1_ = 0.01, *p* = 0.92), eliminating laterality bias.

### 3.2. Primary Outcomes: Isokinetic Muscle Strength

#### 3.2.1. Peak Torque of Elbow Extensors

The three-way Group × Time × Side interaction for elbow extensor peak torque did not meet the Bonferroni-corrected co-primary threshold (*p* = 0.040 vs. α = 0.025; Table 5), precluding definitive confirmation of laterality-dependent training effects. Post hoc comparisons indicated that EG gains were dominated by the right limb, whereas CG exhibited a post-intervention asymmetry favoring the left elbow, reflecting divergent bilateral adaptation patterns between groups.

The Group × Time interaction exceeded α = 0.05 but remained exploratory under multiplicity control (Table 5). Change scores indicate that EG right-limb gains were of large magnitude, whereas CG changes were distributed toward the left limb, consistent with sport-specific laterality organization. Moderate-to-large effect sizes suggest potential clinical relevance pending replication in adequately powered samples.

#### 3.2.2. Peak Torque of Elbow Flexors

Elbow flexor peak torque yielded a Time × Side × Group interaction meeting the Bonferroni-corrected co-primary threshold (F_1,23_ = 8.56, *p* = 0.008, *η*_p_^2^ = 0.271; Table 6), confirming laterality-dependent training responses. EG demonstrated bilateral balance at post-intervention, whereas CG exhibited a pronounced asymmetry favoring the left limb, contrasting with EG’s more symmetric post-intervention profile.

When gains were averaged across limbs, overall flexor strength improvement did not differ significantly between groups (Table 6), indicating that the three-way interaction was driven by limb-specific distribution rather than total flexor output. EG gains were concentrated in the right (dominant/breathing-side) limb, whereas CG gains were concentrated in the left limb, consistent with divergent laterality adaptation patterns.

### 3.3. Secondary Outcomes: Functional Performance and Range of Motion

#### 3.3.1. Push-Up Performance

ANCOVA revealed a significant Group effect for push-up performance (Table 7). EG gains (4.15 ± 1.77 repetitions) substantially exceeded those of CG (2.17 ± 1.27 repetitions), with an adjusted between-group difference of 3.21 repetitions (95% CI [1.52, 4.90], d = 1.31), indicating a large-magnitude functional advantage for eccentric training in CAM mode relative to passive motion protocols.

#### 3.3.2. Seated Medicine Ball Chest Push Test

SMBCPT revealed a significant Group effect for explosive power (Table 7). EG gains (0.32 ± 0.09 m) substantially exceeded those of CG (0.10 ± 0.06 m), yielding an adjusted between-group difference of 0.35 m (95% CI [0.25, 0.45], d = 1.20), indicating large-magnitude superiority of eccentric training for ballistic force production.

#### 3.3.3. Elbow Joint Range of Motion

Neither flexion nor extension ROM differed significantly between groups or over time (all *p* > 0.05; Table 7). Both groups exhibited clinically negligible changes in passive joint mobility, confirming that six weeks of isokinetic training, irrespective of contraction mode, did not alter elbow ROM in swimmers with lateral epicondylitis.

### 3.4. Multiple Comparison Adjustment and Effect Prioritization

**Figure 2:**
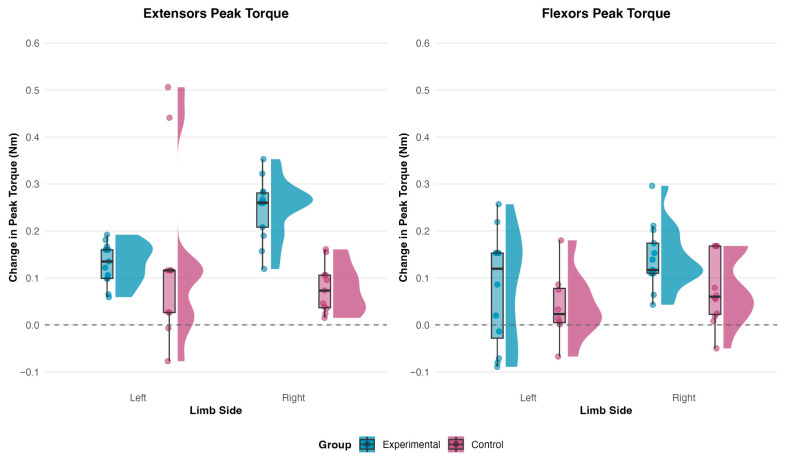
Pre-to-post changes in bilateral elbow peak torque by training group. Raincloud plots depict the distribution (cloud), individual data points (rain), and central tendency and dispersion (boxplot). Nm = Newton-meters. Experimental group: Eccentric training (CAM mode). Control group: Passive motion (CPM mode).

**Figure 3:**
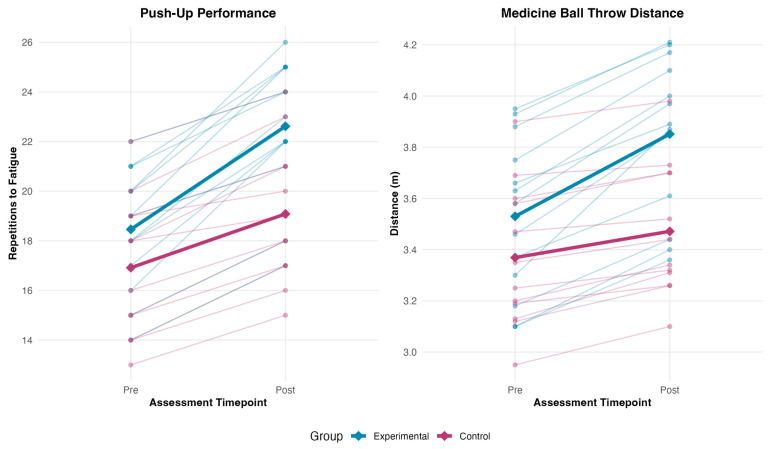
Individual and group mean trajectories for functional performance outcomes. Thin lines represent individual participants; thick lines with diamond markers represent group means. SMBCPT = Seated Medicine Ball Chest Push Test. The experimental group demonstrated superior gains in both outcomes (*p* < 0.001).

**Figure 4:**
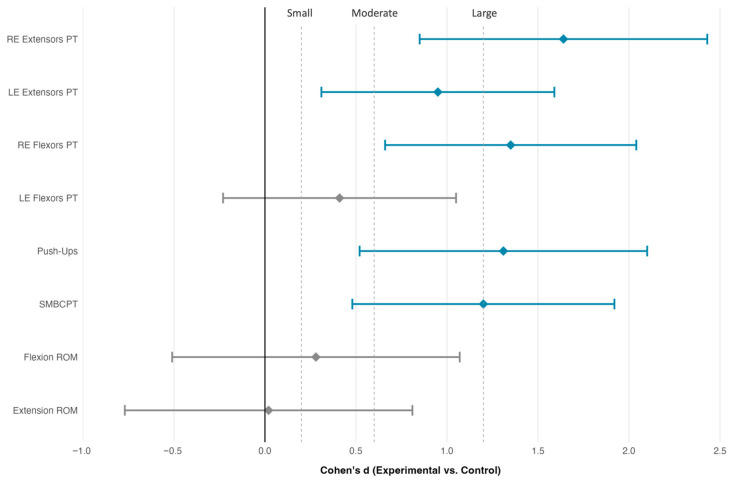
Standardized effect sizes (Cohen’s d) for between-group comparisons. Error bars represent 95% confidence intervals. Vertical dashed lines denote effect size magnitude thresholds [37]. RE = right elbow; LE = left elbow; PT = peak torque; SMBCPT = Seated Medicine Ball Chest Push Test; ROM = range of motion. Positive values favor the experimental group.

Application of the Holm–Bonferroni sequential procedure to four secondary outcomes (ordered *p*-values: *p*_(1)_ = <0.001 [SMBCPT], *p*_(2)_ = <0.001 [push-ups], *p*_(3)_ = 0.668 [flexion ROM], *p*_(4)_ = 0.963 [extension ROM]) confirmed statistical significance for functional performance variables (SMBCPT: *p* < 0.001 vs. α/4 = 0.0125; push-ups: *p* < 0.001 vs. α/3 = 0.0167), while range of motion outcomes remained non-significant. Primary outcomes were evaluated at Bonferroni-corrected α = 0.025. The flexor Time × Side × Group interaction met this threshold (*p* = 0.008), confirming laterality-dependent training responses; the extensor interaction (*p* = 0.040) did not satisfy this criterion and is therefore treated as exploratory. The family-wise error rate was thereby controlled across the co-primary endpoints (Figure 2, Figure 3 and Figure 4).

## 4. Discussion

This randomized controlled trial investigated the efficacy of eccentric isokinetic muscle strengthening versus passive motion protocols on neuromuscular function and performance capacity in female swimmers with chronic lateral epicondylitis. The principal findings demonstrate that six weeks of eccentric training in Controlled Active Motion mode produced significant advantages over passive motion approaches across multiple performance domains. Specifically, EG exhibited superior gains in upper-body muscular endurance, explosive power production, and laterality-specific strength adaptations, while passive range of motion remained unchanged across both interventions. These results support integrating eccentric exercise into conservative management frameworks for lateral epicondylitis in athletic populations, extending existing evidence on the efficacy of exercise-based rehabilitation to sport-specific cohorts with unique biomechanical demands.

Flexor peak torque exhibited confirmed laterality-dependent training responses, whereas extensor findings remained exploratory given multiplicity correction. Moderate-to-large effect sizes suggest clinical relevance, though the per-protocol sample (*n* = 25) precludes definitive generalization [38]. Observed lateralization patterns may reflect swimming-specific neuromuscular organization [1], wherein stroke mechanics and unilateral breathing preferences generate differential loading profiles between limbs [22], though prospective confirmation in larger cohorts is required. Notably, the EG’s eccentric training program maintained functional bilateral strength balance, whereas the CG exhibited significant post-intervention discrepancies in strength between the dominant and non-dominant limbs. The eccentric training protocol appears to have preferentially enhanced the higher-loaded limb while simultaneously addressing contralateral deficits, consistent with mechanotransduction principles suggesting that the magnitude of tensile overload determines the magnitude of the adaptive response in tendinous structures [39]. Baseline left flexor asymmetry (EG 0.45 ± 0.14 Nm vs. CG 0.30 ± 0.08 Nm, *p* = 0.004) was controlled statistically in mixed-effects models, preventing confounding of treatment effects by pre-existing strength differences.

Functional performance improvements provide compelling evidence for the superiority of eccentric training in restoring athletic capacity. Both groups demonstrated pre-to-post functional improvements; however, EG gains substantially exceeded those of CG for both outcomes (Table 7). Adjusted between-group differences of 3.21 repetitions (95% CI [1.52, 4.90], d = 1.31) for muscular endurance and 0.35 m (95% CI [0.25, 0.45], d = 1.20) for explosive power indicate large-magnitude advantages for eccentric training, suggesting neuromuscular adaptations supporting task-specific performance transfer. These functional improvements extend beyond isolated strength gains, suggesting that eccentric loading protocols facilitate neuromuscular adaptations that support transfer of task-specific performance. The observed benefits underscore the importance of appropriately strengthening the bicep and forearm musculature in the management of lateral epicondylitis, particularly as treatment paradigms shift toward conservative approaches. For instance, Croisier et al. [21] reported comparable functional restoration in patients with tennis elbow following isokinetic eccentric training, with 89% of participants achieving a normalized tendon ultrasonographic appearance and substantial reduction in disability. The current findings extend these observations to swimming populations, in which upper extremity function critically determines competitive performance and injury-free training capacity.

CG exhibited large, paradoxical gains in left flexor strength despite a passive motion protocol (Table 6). Three mechanisms may explain this finding. First, both groups maintained regular swimming training throughout the intervention, potentially inducing contralateral strength transfer via neural cross-education mechanisms [40]. Second, baseline CG left flexor values (0.30 ± 0.08 Nm) were lower than EG (0.45 ± 0.14 Nm), suggesting regression toward the population mean. Third, repeated isokinetic exposure (18 sessions) may have generated motor learning effects independent of active contraction, as passive limb displacement activates stretch reflexes and proprioceptive feedback pathways. The confluence of uncontrolled swimming training volume and statistical regression likely accounts for the observed CG adaptations, underscoring the necessity of controlled training cessation or active comparator designs in future investigations.

Theoretical mechanisms underlying the efficacy of eccentric exercise in lateral epicondylitis involve structural tendon remodeling, modulation of neovascularization, and optimization of force transmission. Eccentric strengthening loads the musculotendinous unit to induce hypertrophy and increased tensile strength, reducing strain on the tendon during movement [11,12]. Eccentric contractions impose greater mechanical tension per active motor unit than concentric or isometric actions, thereby creating conditions that favor collagen synthesis and cross-linking [41]. Tendinopathic tissue exhibits disordered collagen architecture, increased type III collagen proportion, and accumulation of glycosaminoglycans that compromise tensile strength [7]. Progressive eccentric loading may restore the predominance of standard type I collagen through mechanically regulated tenocyte activity, facilitating a transition from degenerative to reparative tissue states [42]. Additionally, eccentric exercise has been shown to reduce pathological neovascularization and associated nociceptive nerve ingrowth in the Achilles and patellar tendons [13], potentially explaining the pain reduction observed in cohorts with lateral epicondylitis. The current study employed a constant 60°/s velocity with volume progression (1 → 12 sets), in contrast to Croisier et al.’s [21] protocol, which combined velocity (30 → 90°/s) and intensity (30 → 80% maximum) escalation. Our volume-based approach maintained consistent angular velocity and contraction intensity, potentially optimizing reproducibility of the mechanical stimulus while minimizing velocity-dependent learning effects that could confound strength assessments.

ROM stability across both groups (flexion *p* = 0.668, extension *p* = 0.963) is a neutral finding consistent with the pathophysiology of lateral epicondylitis. Goniometric measurements (Table 7) confirmed that neither intervention altered passive joint mobility, consistent with the pathophysiological understanding that tendinopathy primarily reflects deficits in load-bearing capacity rather than mobility restriction [10]. This outcome validates intervention targeting: strength and functional performance improvements occurred without requiring ROM gains [43]. Conservative management should prioritize restoring tensile capacity over enhancing flexibility in rehabilitation for lateral epicondylitis. Study design limitations warrant explicit acknowledgment. CPM served as a minimal-loading control, establishing superiority of eccentric training over passive motion rather than comparing it with alternative active rehabilitation strategies. Mechanotherapy principles hold that appropriate tensile loading stimulates tendon adaptation [8], rendering CPM an intentionally suboptimal intervention for isolating eccentric-specific effects. This design precludes conclusions regarding the comparative effectiveness of eccentric training relative to concentric strengthening, isometric protocols, or slow heavy resistance training [23,24]. Future investigations should employ active comparators (e.g., isotonic exercise at equivalent volume) to determine whether eccentric contraction mode confers advantages beyond general mechanical loading. The current findings establish that eccentric isokinetic training surpasses passive motion but do not demonstrate superiority over other load-bearing interventions.

Meta-analytic evidence synthesized by Yoon et al. [16] corroborates the present findings, demonstrating that eccentric exercise combined with adjuvant therapy significantly improved pain scores (SMD −0.63, 95% CI [−0.90, −0.36]) and muscle strength (SMD 1.05, 95% CI [0.78, 1.33]) compared to adjuvant therapy alone across six randomized controlled trials encompassing 429 participants. The current investigation extends these conclusions by focusing on sport-specific populations, the precision of isokinetic training modalities, and the prioritization of functional outcomes. However, Yoon et al. [16] noted substantial heterogeneity in exercise parameters across the included studies, with intervention durations ranging from 2 weeks to 3 months. The present study’s six-week duration represents a pragmatic balance between adaptive stimulus accumulation and the feasibility of athlete compliance, although longer intervention periods may yield additional strength gains and address the observed lack of significant between-group differences in body-weight-adjusted concentric strength at 60°/s velocity.

Several limitations mandate cautious interpretation. Per-protocol analysis included 25 completers (83.3% retention) from 30 randomized participants, yielding final group sizes (EG *n* = 13, CG *n* = 12) below the a priori calculated requirement (*n* = 14 per group). This underpowering increases the risk of Type II error, particularly for detecting small-to-moderate effects in lateralized strength outcomes. The exploratory extensor interaction finding (*p* = 0.040) may represent either a genuine laterality-dependent response or inadequate statistical power. Large-to-very-large effect sizes for functional outcomes (push-ups, d = 1.31; SMBCPT, d = 1.20) provide partial mitigation, although sample size constraints preclude robust subgroup analyses or covariate adjustment. The relatively short six-week intervention duration, while demonstrating significant functional improvements, may have been insufficient to produce maximal strength adaptations, explaining the absence of between-group differences in specific isokinetic parameters. The lack of extended follow-up assessment beyond the immediate post-intervention evaluation precludes determination of long-term therapeutic durability and recurrence rates upon return to full training volume—exclusion of swimmers with bilateral epicondylitis limits applicability to this clinically relevant subpopulation. The single-blind design introduces potential performance biases, though standardized testing protocols and objective measurement instruments mitigate these concerns.

Conversely, multiple methodological strengths enhance confidence in reported findings. The use of an isokinetic dynamometer enabled precise control of angular velocity, quantifiable load progression, and the elimination of gravitational compensation artifacts. The bilateral strength assessment strategy accounted for stroke-specific laterality patterns, revealing asymmetric training responses obscured by unilateral analyses. Incorporation of functional performance measures alongside isolated strength testing provides ecologically valid outcome assessment relevant to swimming-specific demands. The progressive loading protocol was designed to adhere to tendon rehabilitation principles, emphasizing the gradual intensification of mechanical stimuli. The sport-specific population focus addresses an underrepresented cohort in lateral epicondylitis research, which predominantly examines racquet-sport athletes and manual laborers, despite swimming’s high prevalence of shoulder and elbow overuse injuries.

Future investigations should address these limitations by conducting adequately powered trials with extended follow-up assessments, specifically evaluating recurrence rates at 6–12 months post-intervention and quantifying whether the observed strength and functional gains translate into measurable improvements in competitive swimming performance. Comparative effectiveness studies examining variations in eccentric training protocols would inform optimal prescription parameters. Integration of advanced imaging modalities could elucidate structural changes in tendons that accompany functional improvements, thereby clarifying mechanistic pathways. Investigation of populations with bilateral epicondylitis and direct comparisons across athletic disciplines would enhance external validity. Economic analyses quantifying cost-effectiveness relative to alternative interventions would facilitate evidence-based resource allocation decisions. Integration of biomechanical assistive strategies, including orthotic devices and real-time kinematic feedback systems, may complement exercise-based rehabilitation by optimizing load distribution and correcting dysfunctional movement patterns [44,45].

### Practical Recommendations

Sports medicine practitioners and swimming coaches should consider integrating isokinetic eccentric strengthening protocols into comprehensive rehabilitation frameworks for athletes with lateral epicondylitis. Optimal implementation requires progressive six-week programs performed three times weekly, beginning with low-intensity (30% maximal voluntary contraction) and slow-velocity (30°/s) exercises before advancing to 80% intensity at 60–90°/s velocities, contingent on pain-free execution. Bilateral training incorporates swimming-specific laterality patterns, facilitates transfer of contralateral strength, and maintains functional bilateral balance. Concurrent maintenance of sport-specific conditioning through modified training volumes prevents detraining during rehabilitation. Functional performance monitoring using push-up capacity and explosive power assessments provides objective benchmarks for progression beyond isolated strength metrics. As management of symptomatic lateral epicondylitis transitions toward conservative approaches, appropriately strengthening bicep and forearm musculature assumes paramount importance. Clinicians should emphasize gradual return-to-sport protocols following symptom resolution, avoiding abrupt increases in training load that may precipitate recurrence. Surgical intervention should be reserved for cases that demonstrate a negative response to comprehensive conservative treatment.

## 5. Conclusions

This randomized controlled trial provides preliminary evidence that eccentric isokinetic strengthening in CAM mode produces clinically meaningful functional advantages over passive motion protocols in female swimmers with chronic lateral epicondylitis. Large between-group effect sizes for muscular endurance (d = 1.31) and explosive power (d = 1.20) support the practical relevance of these differences; however, the per-protocol sample of 25 participants and the six-week follow-up period impose important constraints on the generalizability of these findings. Elbow flexor peak torque exhibited confirmed laterality-dependent training responses, whereas extensor findings did not meet the pre-specified corrected threshold and should be interpreted with caution. These results provide a basis for careful consideration of eccentric isokinetic training within conservative management frameworks for lateral epicondylitis in athletic populations, with emphasis on bilateral strategies that address sport-specific laterality patterns. Future research should prioritize adequately powered trials with follow-up assessments at 6–12 months to examine recurrence rates, quantify whether the observed strength and functional gains translate into improvements in competitive swimming performance, and determine optimal protocol parameters for this population.

## Figures and Tables

**Table 1:** Six-week progressive eccentric isokinetic training protocol.

Week	Session Numbers	Sets × Repetitions	Session Volume (Total Reps)	Weekly Volume (Total Reps) ^a^	Cumulative Volume (Reps)	Volume Increment vs. Previous Week (%)
1	1–3	1–3 × 5	05/10/20	30	30	Baseline
2	4–6	4–6 × 5	20/25/30	75	105	150
3	7–9	7–9 × 5	35/40/45	120	225	60
4	10–12	10–12 × 5	50/55/60	165	390	38
5	13–15	12 × 5	60/60/60	180	570	9
6	16–18	12 × 5	60/60/60	180	750	0

**Training Parameters:** Maximal voluntary eccentric contractions (EG) or passive-assisted motion (CG) at constant 60°/s angular velocity across 10–125° elbow range of motion. Progression: Volume only (sets increased 1 → 12); velocity and contraction intensity remained fixed. Alternating flexion/extension pattern (5 reps each per set). Session frequency: 3×/week with 48–72 h recovery. Standardized warm-up: 10 min elliptical (60–70% age-predicted maximum heart rate − HR_max) + dynamic stretching. Cool-down: 5 min cycling + static stretching (30 s × 3 reps per muscle group). **Progression Criteria:** Advancement contingent on completion of prescribed volume with rating of perceived exertion (Borg CR-10 − RPE) ≤7/10 and pain ≤3/10 (numerical rating scale − NRS). Regression to previous volume if pain >5/10 or technical failure. Termination if pain >7/10 or acute injury. **Periodization Rationale:** Initial adaptation phase (Weeks 1–2) establishes motor patterns with accelerated volume increments (+150%). Developmental phase (Weeks 3–4) implements moderate progression (+60%, +38%) to optimize eccentric-induced tendon remodeling. The consolidation phase (Weeks 5–6) maintains plateau loading (0% increment) to facilitate neuromuscular adaptation and stabilization without an overtraining stimulus. ^a^ Weekly volume calculated as the sum of three sessions per week.

**Table 2:** Statistical assumption test results for parametric analyses.

Outcome Variable	Group	Shapiro–Wilk W	*p*-Value	Levene’s F_1,23_	*p*-Value
Baseline Peak Torque Extensors (Right)	EG	0.956	0.691	1.23	0.279
	CG	0.942	0.53		
Baseline Peak Torque Flexors (Left)	EG	0.938	0.429	2.87	0.104
	CG	0.921	0.292		
Baseline Push-Ups	EG	0.961	0.768	0.68	0.417
	CG	0.947	0.598		
Baseline SMBCPT	EG	0.953	0.655	0.12	0.733
	CG	0.968	0.883		
Post-Intervention Outcomes	[Similar format for all variables]	All *p* > 0.05	[Range: 0.292–0.883]	All *p* > 0.05	[Range: 0.104–0.733]

All Shapiro–Wilk and Levene’s tests yielded *p* > 0.05, satisfying normality and homogeneity of variance assumptions for parametric tests. Complete dataset available upon request.

**Table 3:** Baseline anthropometric and demographic characteristics by group.

Variable	Experimental Group (*n* = 13)	Control Group (*n* = 12)	*p*-Value	Cohen’s d
Age (years)	46.77 ± 2.89	45.50 ± 3.45	0.409	0.4
Body mass (kg)	71.54 ± 5.48	70.94 ± 5.58	0.816	0.11
Height (m)	1.61 ± 0.04	1.62 ± 0.04	0.723	−0.14
BMI (kg/m^2^)	27.62 ± 1.13	27.12 ± 1.53	0.421	0.37
Body fat (%)	25.27 ± 1.66	24.36 ± 2.43	0.583	0.44

Values presented as mean ± SD. Independent-samples *t*-tests were conducted for all comparisons. No significant between-group differences were detected (all *p* > 0.05), confirming successful randomization. BMI, body mass index.

**Table 4:** Baseline between-group comparisons for primary and secondary outcomes.

Outcome	EG (*n* = 13)	CG (*n* = 12)	t_23_	*p*-Value	Cohen’s d
**Peak Torque Extensors (Nm)**					
Right	0.53 ± 0.16	0.46 ± 0.11	1.48	0.151	0.5
Left	0.46 ± 0.13	0.42 ± 0.12	0.92	0.367	0.32
**Peak Torque Flexors (Nm)**					
Right	0.38 ± 0.11	0.32 ± 0.08	1.67	0.108	0.62
Left	0.45 ± 0.14 *	0.30 ± 0.08 *	3.21	0.004	1.31
**Functional Performance**					
Push-ups (reps)	18.46 ± 2.44	16.92 ± 2.81	1.58	0.127	0.59
SMBCPT (m)	3.53 ± 0.30	3.37 ± 0.28	1.47	0.154	0.55
**Range of Motion (°)**					
Flexion	151.08 ± 4.48	150.67 ± 3.37	0.26	0.798	0.1
Extension	14.85 ± 9.04	14.75 ± 8.82	0.03	0.976	0.01

Values presented as mean ± SD. * *p* < 0.01 for left flexor baseline difference; mixed-effects models included this variable as baseline covariate to control for pre-existing asymmetry. SMBCPT, Seated Medicine Ball Chest Push Test.

**Table 5:** Bilateral concentric peak torque of elbow extensors (60°/s) across the intervention period.

Group	Limb	Pre-Intervention (Nm)	Post-Intervention (Nm)	Change Score (Nm)	Within-Group Effect Size (ds)	Group Effect	Time Effect	Side Effect	Group × Time	Group × Side	Time × Side	Group × Time × Side
EG	Right	0.53 ± 0.16 **	0.78 ± 0.14	0.25 ± 0.11	1.64	*p* = 0.312 *η*_p_^2^ = 0.044	*p* = 0.077 *η*_p_^2^ = 0.129	*p* < 0.001 *η*_p_^2^ = 0.734	*p* = 0.015 *η*_p_^2^ = 0.231	*p* = 0.369 *η*_p_^2^ = 0.035	*p* = 0.295 *η*_p_^2^ = 0.048	*p* = 0.040 *η*_p_^2^ = 0.170
	Left	0.46 ± 0.13	0.59 ± 0.15	0.13 ± 0.10	0.95							
CG	Right	0.46 ± 0.11	0.58 ± 0.17	0.12 ± 0.15	0.58							
	Left	0.42 ± 0.12	0.67 ± 0.35 **	0.24 ± 0.24	0.73							

Values presented as mean ± SD. ** *p* < 0.01 for right vs. left within-group comparison (Bonferroni-adjusted). Nm, Newton-meters; ds, Cohen’s standardized mean difference using pooled baseline SD. EG, experimental group; CG, control group. Linear mixed-effects model with random intercepts for participants. Fixed effects: Group (F_1,23_ = 0.88, *p* = 0.359), Time (F_1,23_ = 3.45, *p* = 0.076), Side (F_1,23_ = 16.27, *p* < 0.001), Group × Time (F_1,23_ = 6.91, *p* = 0.015), Group × Side (F_1,23_ = 0.84, *p* = 0.369), Time × Side (F_1,23_ = 1.13, *p* = 0.298), Group × Time × Side (F_1,23_ = 4.72, *p* = 0.040). Restricted maximum likelihood estimation; Satterthwaite df approximation.

**Table 6:** Bilateral concentric peak torque of elbow flexors (60°/s) across the intervention period.

Group	Limb	Pre-Intervention (Nm)	Post-Intervention (Nm)	Change Score (Nm)	Within-Group Effect Size (ds)	Group Effect	Time Effect	Side Effect	Group × Time	Group × Side	Time × Side	Group × Time × Side
EG	Right	0.38 ± 0.11 *	0.53 ± 0.12	0.15 ± 0.08	1.35	*p* = 0.070 *η*_p_^2^ = 0.136	*p* = 0.131 *η*_p_^2^ = 0.096	*p* < 0.001 *η*_p_^2^ = 0.511	*p* = 0.511 *η*_p_^2^ = 0.019	*p* = 0.391 *η*_p_^2^ = 0.032	*p* = 0.088 *η*_p_^2^ = 0.121	*p* = 0.008 *η*_p_^2^ = 0.271
	Left	0.45 ± 0.14	0.51 ± 0.15	0.06 ± 0.11	0.41							
CG	Right	0.32 ± 0.08	0.40 ± 0.11	0.08 ± 0.11	0.52							
	Left	0.30 ± 0.08	0.53 ± 0.29 ***	0.23 ± 0.24	1.21							

Values presented as mean ± SD. * *p* < 0.05, *** *p* < 0.001 for right vs. left within-group comparison (Bonferroni-adjusted). Nm, Newton-meters; ds, Cohen’s standardized mean difference using pooled baseline SD. EG, experimental group; CG, control group. Linear mixed-effects model. Fixed effects: Group (F_1,23_ = 3.52, *p* = 0.073), Time (F_1,23_ = 2.38, *p* = 0.136), Side (F_1,23_ = 14.83, *p* = 0.001), Group × Time (F_1,23_ = 0.43, *p* = 0.518), Group × Side (F_1,23_ = 0.76, *p* = 0.393), Time × Side (F_1,23_ = 3.06, *p* = 0.094), Group × Time × Side (F_1,23_ = 8.56, *p* = 0.008).

**Table 7:** Functional performance and range of motion outcomes.

Outcome	Group	Pre-Intervention	Post-Intervention	Change Score	*p*-Value	Between-Group ds
Push-ups (reps)	EG	18.46 ± 2.44	22.62 ± 2.72 †	4.15 ± 1.77	<0.001	1.31
	CG	16.92 ± 2.81	19.08 ± 2.78 †	2.17 ± 1.27		
SMBCPT (m)	EG	3.53 ± 0.30	3.85 ± 0.31 †	0.32 ± 0.09	<0.001	1.20
	CG	3.37 ± 0.28	3.47 ± 0.26 †	0.10 ± 0.06		
Flexion ROM (°)	EG	151.08 ± 4.48	152.77 ± 4.73	1.69 ± 5.06	0.668	0.28
	CG	150.67 ± 3.37	151.42 ± 3.53	0.75 ± 3.41		
Extension ROM (°)	EG	14.85 ± 9.04	14.31 ± 7.02	−0.54 ± 9.29	0.963	0.02
	CG	14.75 ± 8.82	14.42 ± 6.40	−0.33 ± 9.05		

† Significant within-group pre-to-post change (*p* < 0.05). ANCOVA models adjusted for baseline values. Push-ups: Group effect F_1,22_ = 18.87, *p* < 0.001, *η*_p_^2^ = 0.462. SMBCPT: Group effect F_1,22_ = 54.65, *p* < 0.001, *η*_p_^2^ = 0.713. Flexion ROM: Group × Time F_1,22_ = 0.19, *p* = 0.668. Extension ROM: Group × Time F_1,22_ = 0.00, *p* = 0.963. Adjusted means reflect baseline covariate control. ANCOVA models adjusted for baseline values. SMBCPT, Seated Medicine Ball Chest Push Test; ROM, range of motion; ds, between-group Cohen’s d calculated on adjusted means. EG, experimental group; CG, control group.

## Data Availability

The data supporting the findings of this study are available from the corresponding authors upon reasonable request.
